# Patterns and Motivations of Topical Steroid Use for Skin Whitening in Jazan, Saudi Arabia: A Cross-Sectional Survey

**DOI:** 10.7759/cureus.68455

**Published:** 2024-09-02

**Authors:** Sultan A Jaafari, Radwan Abu Taleb, Mohammed E Mojiri, Osama A Suwaid, Osama A Mobarki, Sarah A Daghriri, Mohammed H Matari, Abdulmajeed A Jadah, Samar F Alhajri, Sara F Alhajri, Sereen D AlQarni, Amani A Mosleh

**Affiliations:** 1 Dermatology, King Fahd Central Hospital, Jazan, SAU; 2 Dermatology, Jazan General Hospital, Jazan, SAU; 3 College of Medicine, Jazan University, Jazan, SAU; 4 College of Medicine, Almaarefa University, Riyadh, SAU; 5 College of Medicine, Alfaisal University, Riyadh, SAU; 6 College of Medicine, King Khalid University, Abha, SAU

**Keywords:** public education, cosmetic use, demographics, motivations, cross-sectional survey, saudi arabia, skin whitening, topical steroids

## Abstract

Background

The use of topical steroids for skin whitening is prevalent in many regions, including Saudi Arabia. This study aims to analyze the patterns, motivations, and demographic factors associated with the use of topical steroids for cosmetic purposes in Jazan, Saudi Arabia.

Methods

This cross-sectional survey was conducted online over three weeks. A structured questionnaire was distributed to adults residing in Jazan, collecting data on demographics, topical steroid use, motivations, and product sources. Descriptive statistics were used to analyze the data.

Results

Among the 340 participants, 173 (50.9%) reported using topical steroids for skin whitening. The majority of users were female (149, 43.8%) and aged between 20 and 30 years (78, 22.9%). Most participants had used topical steroids for less than a year (127, 73.4%), with usage predominantly in the evening (86, 49.7%). Topical steroids were primarily purchased from pharmacies (70, 40.5%), with significant monthly expenditure variability: 55 participants (31.8%) spent between 50 and 110 SAR, and 62 (35.7%) spent more than 150 SAR. Motivations for use included a preference for lighter skin (49, 28.4%) and treatment of melasma (42, 24.3%). Recommendations from friends (71, 41.0%) and TV advertisements (34, 19.8%) influenced product choice.

Conclusion

Topical steroid use for skin whitening is widespread among adults in Jazan, with a notable emphasis on cosmetic outcomes and substantial financial investment. There is a critical need for increased public education on the risks associated with topical steroids and enhanced professional guidance to promote safer usage practices.

## Introduction

Topical steroids are widely used for their anti-inflammatory properties in the treatment of various dermatological conditions. Despite their therapeutic benefits, there is growing concern about their misuse, particularly in the context of skin whitening [1-3]. In many regions, including the Middle East, the use of topical steroids for cosmetic purposes is a prevalent practice, often driven by cultural and social factors that prioritize lighter skin tones.

Skin-whitening products, including those containing topical steroids, are marketed and used to achieve a lighter complexion, reduce pigmentation, and treat conditions such as melasma. Melasma is a common skin disorder characterized by dark, irregular patches on sun-exposed areas of the skin [4,5]. It is particularly prevalent among women and can be exacerbated by factors such as pregnancy, hormonal changes, and sun exposure [6-9]. The use of topical steroids for such purposes raises concerns due to potential side effects, including skin thinning, increased susceptibility to infections, and systemic absorption that could lead to more severe complications [7,10].

The misuse of topical steroids for cosmetic purposes often stems from a lack of awareness regarding their potential adverse effects and the widespread availability of these products without appropriate medical guidance [6-8]. In many cases, individuals may not be fully informed about the risks associated with long-term or inappropriate use. This is compounded by marketing strategies that emphasize the immediate benefits of skin lightening while downplaying or omitting potential risks.

In Saudi Arabia, as in many other countries, there is a significant demand for skin whitening products, and topical steroids are among the substances used. However, comprehensive data on the extent of this practice, its demographic determinants, and the associated risks are limited. Understanding the prevalence and patterns of topical steroid use for skin whitening is crucial for developing effective public health strategies, educational campaigns, and regulatory measures aimed at mitigating the risks associated with these products [9,10].

This study seeks to address these gaps by investigating the use of topical steroids for skin whitening among adults in Jazan, Saudi Arabia. By examining demographic factors, usage patterns, and motivations behind the use of these products, the study aims to provide valuable insights into the scope of the issue inform future interventions to promote safer practices, and raise awareness about the potential risks associated with topical steroid use for cosmetic purposes.

## Materials and methods

Study design

This cross-sectional survey was conducted to assess the use of topical steroids for skin whitening among adults in Jazan, Saudi Arabia. The study utilized convenience sampling to recruit participants from various community settings within the region, aiming to reflect a diverse and representative sample of the local population.

Study participants

To participate in the study, individuals had to be adults aged 18 years or older and able to provide informed consent. Those with cognitive impairments or severe language barriers that could hinder their understanding of or ability to complete the questionnaire were excluded. The recruitment process was carried out online over three weeks, which facilitated broad reach and allowed for convenient access for potential respondents.

Questionnaire development

A structured questionnaire was developed to collect comprehensive data on topical steroid use and related factors [5-10]. The questionnaire encompassed several key areas. It gathered demographic information, including gender, age, nationality, residence, marital status, employment status, educational level, smoking status, and skin color. Information specific to topical steroid use was also collected, covering aspects such as the duration and frequency of use, purchase locations, and monthly expenditure on these products. Additionally, participants were asked about their reasons for using topical steroids, such as cosmetic preferences or medical conditions like melasma, and the sources from which they received recommendations, including friends, advertisements, or healthcare professionals.

Before distribution, the questionnaire underwent pre-testing with a small sample to ensure its clarity and reliability. The final version was administered online to the study population, which allowed participants to complete the survey at their convenience and in their preferred language, Arabic.

Data collection and data analysis

The online questionnaire was distributed over three weeks. This method was selected to provide flexibility for participants, enabling them to respond at a time that suited them best. The online format also ensured that the survey was accessible to a wide audience within the region.

Data were analyzed using SPSS software, version 25.0 (IBM Corp., Armonk, NY, US). Descriptive statistics were computed to summarize the participants' demographic characteristics and their use of topical steroids. Frequencies and percentages were calculated for categorical variables. The results were systematically organized and presented in tables to offer a clear and comprehensive overview of the findings.

## Results

Study population characteristics

Among the 340 participants in this study, 173 (50.9%) reported using topical steroids for skin whitening while 167 (49.1%) had never used these products. The gender distribution among those who used topical steroids was 24 males (7.1%) and 149 females (43.8%). The characteristics of the study population are summarized in Table 1.

**Table 1:** Demographic and socioeconomic characteristics of the study population Data are represented as frequency (n) and percentage (%). Percentages may not sum to 100% due to rounding.

Characteristic		Frequency (n)	Percent (%)
Gender	Male	24	14.4
	Female	149	85.6
Age	Under 20	36	20.8
	20 to 30	78	45.1
	31 to 40	44	25.4
	41 to 50	14	8.1
	Over 50	1	0.6
Nationality	Saudi	169	97.7
	Non-Saudi	4	2.3
Residence	Village	95	54.9
	City	78	45.1
Marital Status	Single	108	62.4
	Married	53	30.6
	Divorced	10	5.8
	Widowed	2	1.2
Employment	Student	84	48.6
	Unemployed	46	27.2
	Retired	1	0.6
	Employed	42	24.6
Education Level	Secondary School/Diploma	39	22.5
	Illiterate	1	0.6
	Below Secondary School	10	5.8
	Postgraduate	2	1.2
	Bachelor’s Degree	56	32.4
	University Student	65	37.9
Smoking	Non-Smoker	154	88.5
	Smoker	15	8.6
	Former Smoker	4	2.3
Skin Color	Black	6	3.5
	Dark Brown	24	13.9
	Light Brown	86	50.0
	Light	57	33.3

Participants were predominantly young adults, with 78 (22.9%) aged between 20 and 30 years. The age distribution showed that 44 participants (12.9%) were between 31 and 40 years, 14 (4.1%) were between 41 and 50 years, and 36 (10.6%) were under 20 years. Only 1 participant (0.3%) was over 50 years old. The majority of participants were Saudi nationals (169, 49.7%), with a small proportion being non-Saudi (4, 1.2%).

Residency was split between those living in a village (95, 27.9%) and those residing in a city (78, 22.9%). Marital status showed that 108 participants (31.8%) were single, 53 (15.6%) were married, 10 (2.9%) were divorced, and 2 (0.6%) were widowed. Employment status included 84 students (24.7%), 46 unemployed individuals (13.5%), 1 retired person (0.3%), and 42 employed individuals (12.4%).

Educational attainment varied among the participants. A total of 56 (16.5%) held a bachelor’s degree, 65 (19.1%) were university students, 39 (11.5%) had completed secondary school or a diploma, and 10 (2.9%) had less than secondary education. Illiteracy was reported by 1 participant (0.3%) while 2 (0.6%) had postgraduate education. Smoking status indicated that 154 participants (45.3%) were non-smokers, 15 (4.4%) were current smokers, and 4 (1.2%) were former smokers. Skin color distribution included 6 participants (1.8%) with black skin, 24 (7.1%) with dark brown skin, 86 (25.3%) with light brown skin, and 57 (16.8%) with light skin.

Topical steroid use characteristics

Among the 173 participants who used topical steroids, the duration of use varied significantly. A majority, 127 participants (73.4%), reported using these products for less than a year while 46 (26.6%) had used them for over a year. The patterns of usage were predominantly in the evening (86, 49.7%), with some participants using the products in the morning and evening (60, 34.7%), and others using them only in the morning (17, 9.8%) or at other times (10, 5.8%) (Table 2).

**Table 2:** Characteristics of topical steroid use Data are represented as frequency (n) and percentage (%). Percentages may not sum to 100% due to rounding. SAR = Saudi Riyal

Characteristic		Frequency (n)	Percent (%)
Duration of Use	Less than a year	127	73.4
	More than a year	46	26.6
Time of Use	Morning	17	9.8
	Morning and Evening	60	34.7
	Evening	86	49.7
	Other Times	10	5.8
Source of Purchase	Pharmacy	70	40.5
	Beauty Stores	22	12.7
	Both	54	31.2
	Other	27	15.6
Monthly Expenditure (SAR)	50-110	55	31.8
	110-150	34	19.7
	Less than 50	22	12.7
	More than 150	62	35.7
Reason for Use	Melasma/Hyperpigmentation	42	24.3
	Preference for Lighter Skin	49	28.4
	Both	37	21.4
	Other	45	25.9
Basis for Choosing Products	Friend’s Advice	71	41.0
	TV Ads	34	19.8
	Pharmacist’s Advice	19	11.0
	Doctor’s Advice	16	9.2
	Other	25	14.5
	Beauty Store Clerk’s Advice	8	4.6

Participants purchased topical steroids primarily from pharmacies (70, 40.5%), with a notable proportion buying from both pharmacies and beauty stores (54, 31.2%), and others from beauty stores only (22, 12.7%). Additionally, 27 participants (15.6%) obtained their products from other sources.

Monthly expenditure on skin whitening products showed a range of spending: 55 participants (31.8%) spent between 50 and 110 SAR, 34 (19.7%) spent between 110 and 150 SAR, 22 (12.7%) spent less than 50 SAR, and 62 (35.7%) spent more than 150 SAR.

The reasons for using topical steroids varied. The primary reasons included a preference for lighter skin (49, 28.4%) and the treatment of melasma or hyperpigmentation (42, 24.3%). Some participants used these products for both reasons (37, 21.4%), while other reasons were cited by 45 participants (25.9%).

Product selection was influenced by advice from friends (71, 41.0%) and TV advertisements (34, 19.8%). Recommendations from pharmacists (19, 11.0%) and doctors (16, 9.2%) were less common. Other sources of information and beauty store clerks contributed to product choice in 25 (14.5%) and 8 (4.6%) cases, respectively.

## Discussion

This study provides a comprehensive analysis of the use of topical steroids for skin whitening among adults in Jazan, Saudi Arabia. The findings highlight significant patterns and characteristics related to the use of these products, offering insights into demographic influences, usage behaviors, and underlying motivations.

The study population comprised a diverse group of adults, with a slight majority (50.9%) reporting the use of topical steroids for skin whitening. Notably, the user group was predominantly female (43.8%) compared to male participants (7.1%), reflecting broader trends in cosmetic product use where women are more frequently targeted by and engage with skin whitening products. The age distribution indicated a younger demographic, with the majority of users aged between 20 and 30 years, aligning with the observed higher cosmetic product use in younger age groups [8,10].

The predominance of Saudi nationals (49.7%) in the study reflects the regional context of the sample. Residents of both urban and rural areas were represented, though urban dwellers were slightly underrepresented. Marital status and educational attainment among participants varied, with a significant proportion being single and holding higher education degrees. These demographic factors may influence perceptions of beauty and skincare practices, contributing to the observed patterns of topical steroid use [11-13].

The analysis revealed diverse patterns in the use of topical steroids. A substantial majority of users (73.4%) had used these products for less than a year, suggesting relatively recent adoption or discontinuation of use. The predominant usage in the evening (49.7%) could reflect recommendations for application to minimize exposure to sunlight, which may exacerbate side effects. The frequency of use, with a considerable number of participants using products in both morning and evening, indicates a strong commitment to achieving desired cosmetic outcomes.

The primary sources for obtaining topical steroids were pharmacies (40.5%), indicating a reliance on established retail channels for purchasing these products. The secondary use of beauty stores and alternative sources suggests a degree of market diversity and consumer choice. The observed expenditure patterns, with significant variability in monthly spending, highlight the financial commitment involved in maintaining skin whitening regimens [10,14].

The reasons for using topical steroids were multifaceted. A preference for lighter skin was the primary motivation for a substantial portion of users while others sought treatment for melasma or hyperpigmentation. This dual motivation underscores the complex interplay between aesthetic preferences and medical needs. The significant number of participants using these products for both cosmetic and therapeutic reasons points to the diverse and sometimes overlapping drivers behind their use [12-15].

Product selection was heavily influenced by recommendations from friends and TV advertisements. This reliance on informal and media-driven advice highlights potential gaps in professional guidance and underscores the need for targeted educational interventions. Recommendations from healthcare professionals, although less frequent, were valued by a smaller subset of users, suggesting an opportunity to enhance the role of medical advice in guiding the safe and informed use of topical steroids [8-10].

The findings of this study underscore the importance of addressing the risks associated with topical steroid use for cosmetic purposes. The high prevalence of use, coupled with varied motivations and significant financial investment, points to a need for greater public awareness regarding potential side effects and safe practices. Educational campaigns should focus on informing users about the risks of prolonged or inappropriate use and promoting alternatives that do not involve corticosteroids [7,9].

This study is subject to several limitations. The reliance on self-reported data may introduce response biases, and the convenience sampling method may not fully capture the diversity of the broader population. Additionally, the online nature of the survey may have excluded individuals with limited internet access, potentially affecting the generalizability of the findings.

## Conclusions

This study reveals that the use of topical steroids for skin whitening is prevalent among adults in Jazan, Saudi Arabia, with significant variation in usage patterns, motivations, and expenditure. The findings highlight a strong preference for lighter skin and underscore the need for increased public awareness regarding the potential risks associated with topical steroid use. The reliance on informal sources of product recommendations suggests a critical need for enhanced educational initiatives and professional guidance to ensure safe usage practices. Addressing these issues through targeted interventions and further research will be essential for promoting informed and responsible use of skin whitening products.

## References

[1] Dey VK (2014). Misuse of topical corticosteroids: a clinical study of adverse effects. Indian Dermatol Online J.

[2] Maneli MH, Wiesner L, Tinguely C (2016). Combinations of potent topical steroids, mercury and hydroquinone are common in internationally manufactured skin-lightening products: a spectroscopic study. Clin Exp Dermatol.

[3] Sommerlad M (2022). Skin lightening: causes and complications. Clin Exp Dermatol.

[4] Shams A, Khan IU, Iqbal H (2016). Analysis of salicylic acid, arbutin and corticosteroids in skin whitening creams available in Pakistan using chromatographic techniques. Int J Cosmet Sci.

[5] Owolabi JO, Fabiyi OS, Adelakin LA, Ekwerike MC (2020). Effects of skin lightening cream agents - hydroquinone and kojic acid, on the skin of adult female experimental rats. Clin Cosmet Investig Dermatol.

[6] Nithya S, Srinivas CR, Lal S (2024). Detection of steroids in topical fairness preparations using histamine wheal test. Indian Dermatol Online J.

[7] Qian W, Liu W, Zhu D, Cao Y, Tang A, Gong G, Su H (2020). Natural skin-whitening compounds for the treatment of melanogenesis (review). Exp Ther Med.

[8] Sinha A, Kar S, Yadav N, Madke B (2016). Prevalence of topical steroid misuse among rural masses. Indian J Dermatol.

[9] Bamerdah S, Alhothali OS, Aldajani BM, Alghanemi L, Mleeh NT (2023). A cross-sectional study of the knowledge, practice, and attitude towards skin-lightening products among the general population in the Western Region of Saudi Arabia. Cureus.

[10] Jacob SM, Thillaikarasi A (2023). Awareness on skin lightening practice and their side effects among nursing staff in a tertiary care centre. Indian J Dermatol.

[11] Yusuf MA, Mahmoud ND, Rirash FR, Stoff BK, Liu Y, McMichael JR (2019). Skin lightening practices, beliefs, and self-reported adverse effects among female health science students in Borama, Somaliland: a cross-sectional survey. Int J Womens Dermatol.

[12] Sendrasoa FA, Ranaivo IM, Andrianarison M, Raharolahy O, Razanakoto NH, Ramarozatovo LS, Rapelanoro Rabenja F (2017). Misuse of topical corticosteroids for cosmetic purpose in Antananarivo, Madagascar. Biomed Res Int.

[13] Mahé A, Ly F, Aymard G, Dangou JM (2003). Skin diseases associated with the cosmetic use of bleaching products in women from Dakar, Senegal. Br J Dermatol.

[14] Pollock S, Taylor S, Oyerinde O (2021). The dark side of skin lightening: an international collaboration and review of a public health issue affecting dermatology. Int J Womens Dermatol.

[15] Mahé A, Perret JL, Ly F, Fall F, Rault JP, Dumont A (2007). The cosmetic use of skin-lightening products during pregnancy in Dakar, Senegal: a common and potentially hazardous practice. Trans R Soc Trop Med Hyg.

